# Differences between human and machine perception in medical diagnosis

**DOI:** 10.1038/s41598-022-10526-z

**Published:** 2022-04-27

**Authors:** Taro Makino, Stanisław Jastrzębski, Witold Oleszkiewicz, Celin Chacko, Robin Ehrenpreis, Naziya Samreen, Chloe Chhor, Eric Kim, Jiyon Lee, Kristine Pysarenko, Beatriu Reig, Hildegard Toth, Divya Awal, Linda Du, Alice Kim, James Park, Daniel K. Sodickson, Laura Heacock, Linda Moy, Kyunghyun Cho, Krzysztof J. Geras

**Affiliations:** 1 Center for Data Science, New York University, New York, NY USA; 2 Department of Radiology, NYU Langone Health, New York, NY USA; 3 Center for Advanced Imaging Innovation and Research, NYU Langone Health, New York, NY USA; 4 Department of Computer Science, Courant Institute, New York University, New York, NY USA; 5 Vilcek Institute of Graduate Biomedical Sciences, NYU Grossman School of Medicine, New York, NY USA; 6 Perlmutter Cancer Center, NYU Langone Health, New York, NY USA; 7 Faculty of Electronics and Information Technology, Warsaw University of Technology, Warszawa, Poland

**Keywords:** Computer science, Statistics, Medical imaging

## Abstract

Deep neural networks (DNNs) show promise in image-based medical diagnosis, but cannot be fully trusted since they can fail for reasons unrelated to underlying pathology. Humans are less likely to make such superficial mistakes, since they use features that are grounded on medical science. It is therefore important to know whether DNNs use different features than humans. Towards this end, we propose a framework for comparing human and machine perception in medical diagnosis. We frame the comparison in terms of perturbation robustness, and mitigate Simpson’s paradox by performing a subgroup analysis. The framework is demonstrated with a case study in breast cancer screening, where we separately analyze microcalcifications and soft tissue lesions. While it is inconclusive whether humans and DNNs use different features to detect microcalcifications, we find that for soft tissue lesions, DNNs rely on high frequency components ignored by radiologists. Moreover, these features are located outside of the region of the images found most suspicious by radiologists. This difference between humans and machines was only visible through subgroup analysis, which highlights the importance of incorporating medical domain knowledge into the comparison.

Following their success in the natural image domain^1–7^, deep neural networks (DNNs) have achieved human-level performance in various image-based medical diagnosis tasks^8–20^. DNNs have a number of additional benefits: they can diagnose quickly, do not suffer from fatigue, and can be deployed anywhere in the world. However, they currently possess a weakness which severely limits their clinical applicability. They cannot be fully trusted, given their tendency to fail for reasons unrelated to underlying pathology. For example, a dermatologist-level skin cancer classifier, approved for use as a medical device in Europe, learned to associate surgical skin markings with malignant melanoma^21^. As a result, the classifier’s false positive rate increased by 40% in an external validation. Also, a pneumonia classifier was found to exploit differences in disease prevalence between sites, and was not making predictions solely based on underlying pathology^22^.

In contrast, humans are more likely to fail because of the difficulty of the task, rather than for a superficial reason. This is partly because humans use features rigorously developed in their respective medical fields. Instead of being merely correlated with the presence of disease, there is a physiological reason such features are predictive. Therefore, in order to establish trust in machine-based diagnosis, it is important to know whether machines use different features than humans. Drawing inspiration from the natural image domain, we perform this comparison with respect to perturbation robustness^23–28^. By removing certain information from the input and analyzing the resulting change in prediction, we can infer the degree to which that information was utilized. We extend this line of work, taking into account a critically important consideration for medical diagnosis.

We argue that subgroup analysis is necessary in order to draw correct conclusions regarding medical diagnosis. In terms of predictive performance, different types of predictive errors can vary greatly in clinical significance^29^. All errors are treated equally when using empirical risk minimization, which can lead to a large disparity in predictive performance across subgroups. Robust optimization addresses this issue by considering the performance of various subgroups, and optimizing the worst-case subgroup performance^30,31^. Additionally, a failure to incorporate subgroups can lead to incorrect conclusions about perception due to Simpson’s paradox^32^. The paradox is that subgroup-specific relationships can disappear or even reverse when the subgroups are aggregated. We therefore propose a framework which uses subgroup-specific perturbation robustness to compare human and machine perception in medical diagnosis. We demonstrate our framework with a case study in breast cancer screening, and show that failure to account for subgroups would indeed result in incorrect conclusions. It is important to note that while we analyze perturbation robustness, our purpose here is not to improve the robustness of machine-based diagnosis. Instead, we use perturbation robustness as a means of comparing human and machine perception.

**Figure 1:**
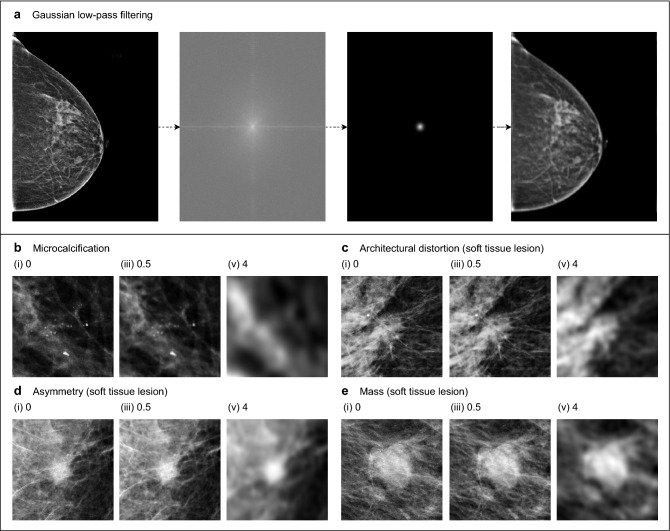
Identification of subgroups and an input perturbation. In our breast cancer screening case study, we separately analyzed two subgroups: microcalcifications and soft tissue lesions, using Gaussian low-pass filtering as the input perturbation. **(a)** Gaussian low-pass filtering is composed of three operations. The unperturbed image is transformed to the frequency domain via the two-dimensional discrete Fourier transform (DFT). A Gaussian filter is applied, attenuating high frequencies. The image is then transformed back to the spatial domain with the inverse DFT. **(b–e)** Gaussian low-pass filtering applied to various types of malignant breast lesions. Subfigures (i–iii) show the effects of low-pass filtering of increasing severity. **(b)** Microcalcifications are tiny calcium deposits in breast tissue that appear as white specks. Radiologists must often zoom in significantly in order to see these features clearly. Since these microcalcifications have a strong high frequency component, their visibility is severely degraded by low-pass filtering. **(c)** Architectural distortions indicate a tethering or indentation in the breast parenchyma. One of their identifying features are radiating thin straight lines, which become difficult to see after filtering. **(d)** Asymmetries are unilateral fibroglandular densities that do not meet the criteria for a mass. Low-pass filtering blurs their borders, making them blend into the background. **(e)** Masses are areas of dense breast tissue. Like asymmetries, masses generally become less visible after low-pass filtering, since their borders become less distinct. In our subgroup analysis, we aggregated architectural distortions, asymmetries, and masses into a single subgroup called “soft tissue lesions.” This grouping was designed to distinguish between localized and nonlocalized lesions. Soft tissue lesions on the whole are far less localized than microcalcifications, and they require radiologists to consider larger regions of the image during the process of diagnosis. Figure created with drawio v13.9.0 https://github.com/jgraph/drawio.

**Figure 2:**
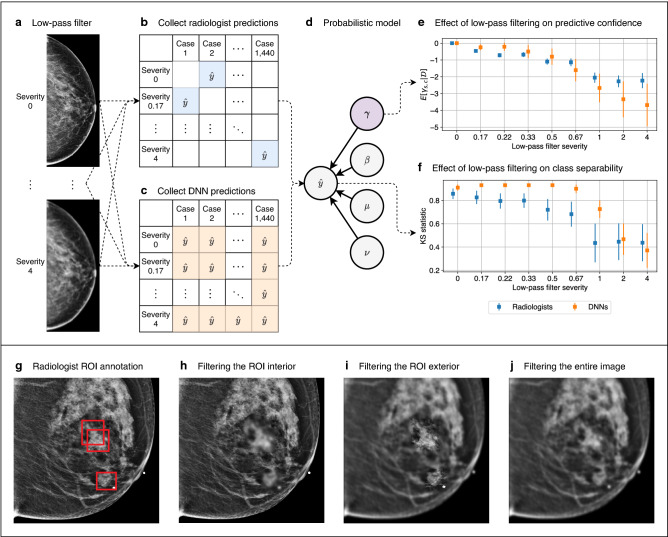
Our framework applied to breast cancer screening. **(a–f)** Comparing radiologists and DNNs with respect to their perturbation robustness. **(a)** We applied low-pass filtering to a set of mammograms using a wide range of filter severities. **(b)** We conducted a reader study in which each reader was provided with the same set of mammograms. Each reader saw each exam once, and each exam was filtered with a random severity. Thus, each radiologist’s predictions populate a sparse matrix. **(c)** Predictions were collected from DNNs on the same set of exams. Unlike radiologists, DNNs made a prediction for all pairs of filter severities and cases, so their predictions form a dense matrix. **(d)** Probabilistic modeling was applied to the predictions, where a latent variable $\gamma$ measures the effect of low-pass filtering, and a separate variable $\eta$ factors out individual idiosyncrasies. **(e)** We examined the posterior expectation of $\gamma$ to evaluate the effect of low-pass filtering on predictive confidence. **(f)** We sampled from the posterior predictive distribution and computed the distance between the distributions of predictions for malignant and nonmalignant cases. This represents the effect that low-pass filtering has on class separability. **(g–j)** Comparison of radiologists and DNNs with respect to the regions of an image they find most suspicious. **(g)** Radiologists annotated up to three regions of interest (ROIs) that they found most suspicious. We then applied low-pass filtering to: **(h)** the ROI interior, **(i)** the ROI exterior, and **(j)** the entire image. We analyzed the robustness of DNNs to these three filtering schemes in order to understand the degree to which the DNNs utilize information in the interiors and exteriors of the ROIs. Figure created with drawio v13.9.0 https://github.com/jgraph/drawio.

In this framework, we identify subgroups that are diagnosed by humans in a significantly different manner, and specify an input perturbation that removes clearly-characterizable information from each of these subgroups. See Fig. 1 for an illustration of this applied to breast cancer screening. Predictions are collected from humans and machines on medical images perturbed with varying severity. We then apply probabilistic modeling to these predictions to capture the isolated effect of the perturbation, while factoring out individual idiosyncrasies. The resulting model is used to compare the perturbation robustness of humans and machines in terms of two criteria that are important for diagnosis: predictive confidence and class separability. Predictive confidence measures the strength of predictions, and is independent of correctness. Class separability represents correctness, and is quantified as the distance between the distributions of predictions for positive and negative cases. If humans and machines exhibit a different sensitivity to this perturbation, it implies that they are using different features. Next, we investigated the degree to which humans and machines agree on the most suspicious regions of an image. Radiologists annotated up to three regions of interest (ROIs) that they found most suspicious, and we analyzed the robustness of DNNs when low-pass filtering is applied to the interiors and exteriors of the ROIs, and to the entire image. See Fig. 2 for a visualization of this procedure for comparing humans and machines in the setting of breast cancer screening.

In our case study, we examined the sensitivity of radiologists and DNNs to Gaussian low-pass filtering, drawing separate conclusions for microcalcifications and soft tissue lesions. Low-pass filtering removes clearly-characterizable information in the form of high frequency components. In order to draw precise conclusions, it is more important for the removed information to be clearly-characterizable than clinically realistic. For example, a change in medical institutions is clinically realistic, but it is unclear what information is being removed. For microcalcifications, we found that radiologists and DNNs are both sensitive to low-pass filtering. Therefore, we could not conclude that humans and DNNs use different features to detect microcalcifications. Meanwhile, for soft tissue lesions, we found that humans are invariant to low-pass filtering, while DNNs are sensitive. This divergence suggests that humans and DNNs use different features to detect soft tissue lesions. Furthermore, using the ROIs annotated by radiologists, we found that a significant proportion of the high frequency components in soft tissue lesions used exclusively by DNNs lie outside of the regions found most suspicious by radiologists. Crucially, we show that without subgroup analysis, we would fail to observe this difference in behavior on soft tissue lesions, thus artificially inflating the similarity of radiologists and DNNs.

## Results

**Experimental setup.** We experimented with the NYU Breast Cancer Screening Dataset^33^ developed by our research team and used in a number of prior studies^12–14,34,35^, and applied the same training, validation, and test set data split as previously reported. This dataset consists of 229,426 screening mammography exams from 141,473 patients. Each exam contains at least four images, with one or more images for each of the four standard views of screening mammography: left craniocaudal (L-CC), left mediolateral oblique (L-MLO), right craniocaudal (R-CC), and right mediolateral oblique (R-MLO). Each exam is paired with labels indicating whether there is a malignant or benign finding in each breast. See Supplementary Information Fig. 1 for an example of a screening mammogram. We used a subset of the test set for our reader study, which is also the same subset used in the reader study of^12^. For our DNN experiments, we used two architectures in order to draw general conclusions: the deep multi-view classifier (DMV)^12^, and the globally-aware multiple instance classifier (GMIC)^13,14^. We primarily report results for GMIC, since it is the more recent and better-performing model. The corresponding results for DMV, which support the generality of our findings, are provided in the Supplementary Information section.

### Perturbation reader study

 In order to compare the perception of radiologists and DNNs, we applied Gaussian low-pass filtering to mammograms, and analyzed the resulting effect on their predictions. We selected nine filter severities ranging from unperturbed to severe, where severity was represented as a wavelength in units of millimeters on the physical breast. Details regarding the calculation of the filter severity are provided in the Methods section. Figure 1 demonstrates how low-pass filtering affects the appearance of malignant breast lesions.

We conducted a reader study in order to collect predictions for low-pass filtered images from radiologists. This reader study was designed to be identical to that of^12^, except that the mammograms were randomly low-pass filtered in our case. We assigned the same set of 720 exams to ten radiologists with varying levels of experience. The images were presented to the radiologists in a conventional format, and an example is shown in Supplementary Information Fig. 1. Each radiologist read each exam once, and for each exam, we uniformly sampled one severity level out of our set of nine, and applied it to all images in the exam. The radiologists made binary predictions indicating the presence of a malignant lesion in each breast. We describe the details of the reader study in the Methods section.

We then trained five DNNs from random weight initializations, and made predictions on the same set of 720 exams. We repeated this nine times, where the set of exams was low-pass filtered with each of the nine filter severities. We note that for each DNN, we made a prediction for every pair of exam and filter severity. In contrast, for each radiologist, we only had predictions for a subset of the possible combinations of exam and filter severity. This means that if we arrange the predictions in a matrix where each row represents a filter severity and each column an exam, the matrix of predictions is sparse for each radiologist, and dense for each DNN. This fact is visualized in Fig. 2b, c. The sparsity of the radiologist predictions is by design; we were careful to ensure that each radiologist only read each exam once, since if they were to have seen the same exam perturbed with multiple filter severities, their predictions would have been unlikely to be independent. However, the sparsity prevents us from comparing radiologists and DNNs using evaluation metrics that use predictions for the complete set of exams. We therefore utilized probabilistic modeling to use the available radiologist predictions to infer values for the missing predictions.

### A probabilistic model of predictions

**Figure 3:**
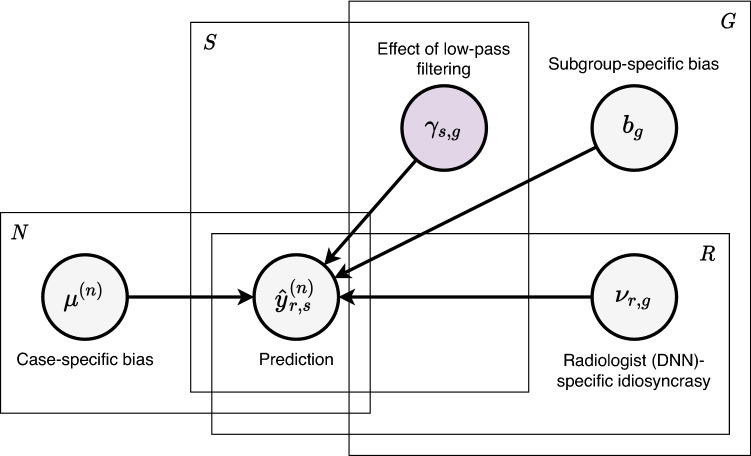
Probabilistic model. Our modeling assumption is that each prediction of radiologists and DNNs is influenced by four latent variables. ${\hat{y}}_{r, s}^{(n)}$ is radiologist (DNN) *r*’s prediction on case *n* filtered with severity *s*. As for the latent variables, $b_g$ represents the bias for subgroup *g*, $\mu ^{(n)}$ is the bias for case *n*, $\gamma _{s, g}$ is the effect that low-pass filtering with severity *s* has on lesions in subgroup *g*, and $\nu _{r, g}$ is the idiosyncrasy of radiologist (DNN) *r* on lesions in subgroup *g*. Our analysis relies on the posterior distribution of $\gamma _{s, g}$, as well as the posterior predictive distribution of ${\hat{y}}_{r, s}^{(n)}$. The other latent variables factor out potential confounding effects. Figure created with drawio v13.9.0 https://github.com/jgraph/drawio.

We applied probabilistic modeling to achieve two purposes. The first is to study the effect of low-pass filtering on specific subgroups of lesions in isolation, after factoring out various confounding effects such as the idiosyncracies of individual radiologists and DNNs. The second is to infer the radiologists’ predictions for each pair of exam and filter severity, since some pairs were missing by design. We modeled the radiologists’ and DNNs’ predictions as i.i.d. Bernoulli random variables. Let us denote radiologist (DNN) *r*’s prediction on case *n* filtered with severity *s* as ${\hat{y}}_{r, s}^{(n)}$. We parameterized our model as

1 $$\begin{aligned} {\hat{y}}_{r, s}^{(n)} \sim {{\,\mathrm{Bernoulli}\,}}(\sigma (b_g + \mu ^{(n)} + \gamma _{s, g} + \nu _{r, g})), \end{aligned}$$

where $\sigma$ is the logistic function. There are four latent variables with the following interpretation: $b_g$ represents the bias of exams in subgroup *g*, $\mu ^{(n)}$ is the bias of exam *n*, $\gamma _{s, g}$ is the effect that low-pass filtering with severity *s* has on exams in subgroup *g*, and $\nu _{r, g}$ is the idiosyncrasy of radiologist (DNN) *r* on exams in subgroup *g*. See Fig. 3 for a graphical representation of our model. We considered several parameterizations of varying complexity, and selected the one with the maximum marginal likelihood. See the Methods section for details regarding our probabilistic model.

### Comparing humans and machines with respect to their perturbation robustness

**Figure 4:**
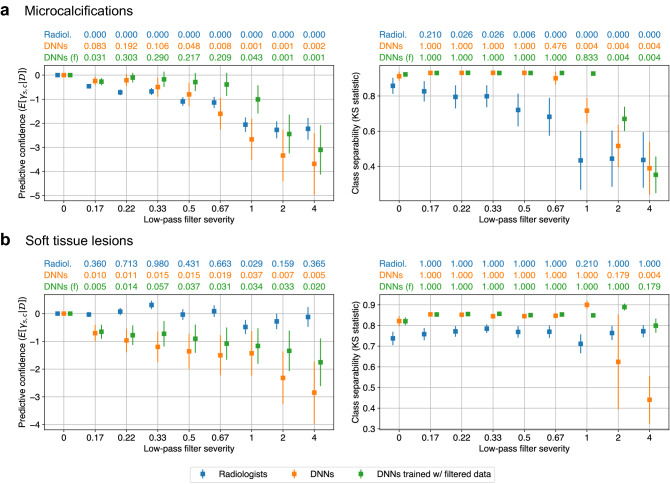
Comparing human and machines with respect to their perturbation robustness. The left subfigures represent the effect on predictive confidence, measured as the posterior expectation of $\gamma _{s, g}$ for severity *s* and subgroup *g*. The values at the top of each subfigure represent the probability that the predictive confidence for each severity is greater than zero. Smaller values for a given severity indicate a more significant downward effect on predictive confidence. The right subfigures correspond to the effect on class separability, quantified by the two-sample Kolmogorov–Smirnov (KS) statistic between the predictions for the positive and negative classes. The values at the top of each subfigure are the *p*-values of a one-tailed KS test between the KS statistics for a given severity and severity zero. Smaller values indicate a more significant downward effect on class separability for that severity. **(a)** For microcalcifications, low-pass filtering degrades predictive confidence and class separability for both radiologists and DNNs. When DNNs are trained with filtered data, the effects on predictive confidence and class separability are reduced, but not significantly. **(b)** For soft tissue lesions, filtering degrades predictive confidence and class separability for DNNs, but has no effect on radiologists. When DNNs are trained with filtered data, the effect on predictive confidence is reduced, and DNN-derived class separability becomes invariant to filtering. Figure created with drawio v13.9.0 https://github.com/jgraph/drawio.

Using the probabilistic model, we compared how low-pass filtering affects the predictions of radiologists and DNNs, separately analyzing microcalcifications and soft tissue lesions. We performed each comparison with respect to two metrics: predictive confidence and class separability. Since the latent variable $\gamma _{s, g}$ represents the effect of low-pass filtering on each prediction, we examined its posterior distribution in order to measure the effect on predictive confidence. We sampled values of ${\hat{y}}_{r, s}^{(n)}$ from the posterior predictive distribution in order to quantify how low-pass filtering affects class separability. We computed the Kolmogorov-Smirnov (KS) statistic between the sampled predictions for the positive and negative class. This represents the distance between the two distributions of predictions, or how separated the two classes are. Sampling from the posterior predictive distribution was necessary for radiologists, since we did not have a complete set of predictions from them. Although such sampling was not strictly necessary for DNNs given the full set of available predictions, we performed the same posterior sampling for DNNs in order to ensure a fair comparison.

Figure 4a presents the results for microcalcifications. We only consider DNNs that are trained with unperturbed data in this section. The results for training DNNs on low-pass filtered data are discussed in the next section. The left subfigure represents predictive confidence, as measured by the posterior expectation of $\gamma _{s, g}$. Since low-pass filtering removes the visual cues of malignant lesions, we hypothesized that it should decrease predictive confidence. In other words, we expected to see ${\mathbb {E}}[\gamma _{s, g} \mid {\mathcal {D}}] \le 0$. Above the left subfigure, we report $P(\gamma _{s, g} > 0 \mid {\mathcal {D}})$ in order to quantify how much the posterior distributions $P(\gamma _{s, g} \mid {\mathcal {D}})$ align with this hypothesis. Small values indicate a significant negative effect on predictive confidence. We note that these values are not intended to be interpreted as the *p*-values of a statistical test. Instead, they quantify the degree to which each $\gamma _{s, g}$ is negative. We observe that for microcalcifications, low-pass filtering decreases the predictive confidence of both radiologists and DNNs. There is, however, a nuanced difference in that for the range of most severe filters, the effect is constant for radiologists, while DNNs continue to become less confident.

The right subfigure of Fig. 4a depicts the effect of low-pass filtering on class separability. This is quantified by the KS statistic between the predictions for the positive and negative class, where the positive class is restricted to malignant microcalcifications. Similar to our hypothesis that low-pass filtering decreases predictive confidence, we hypothesized that it should also reduce class separability. That is, we expected the KS statistics for severity $s > 0$ to be smaller than those for $s = 0$. This is because removing the visual cues for malignant lesions should make it more difficult to distinguish between malignant and nonmalignant cases. We tested this hypothesis using the one-tailed KS test between the KS statistics for $s = 0$ and $s > 0$. The *p*-values for this test are reported above the right subfigures, where small values mean that filtering significantly decreases class separability. We found that low-pass filtering decreases class separability for both radiologists and DNNs, but in different ways. The radiologists’ class separability steadily declines for the range of less severe filters, while it is constant for DNNs. Meanwhile, similar to what we observed for predictive confidence, the radiologists’ class separability is constant for the range of most severe filters, while it continues to decline for DNNs. While there are some nuanced differences, speaking generally, the radiologists and DNNs are both sensitive to low-pass filtering on microcalcifications. Therefore, we cannot conclude that humans and DNNs use different features to detect microcalcifications.

Next, we compared how low-pass filtering affects the predictions of radiologists and DNNs on soft tissue lesions (Fig. 4b). The results show that low-pass filtering degrades the predictive confidence and class separability of DNNs, while having almost no effect on radiologists. Since DNNs are sensitive to a perturbation that radiologists are invariant to, we conclude that humans and DNNs use different features to detect soft tissue lesions. This is a significant difference between human and machine perception, and may be attributable to certain inductive biases possessed by DNNs, such as their tendency to look at texture over shape^36^. Such differences must be better understood and reconciled in order to establish trust in machine-based diagnosis.

### Training DNNs with low-pass filtered data

 We observed that low-pass filtering decreases the predictive confidence and class separability of DNNs for all lesion subgroups. However, since the DNNs only encountered low-pass filtering during testing, it is possible that this effect is solely due to the dataset shift between training and testing. We therefore repeated the previous experiments for DNNs, where the same filtering was applied during both training and testing. We then examined whether the effects of low-pass filtering on the DNNs’ perception could be attributed to information loss rather than solely to dataset shift.

For microcalcifications (Fig. 4a), training on filtered data slightly reduced the effect of low-pass filtering on predictive confidence and class separability, but the effect was still present, particularly for the most severe filters. This is evident from comparing the *p*-values for the two types of DNNs. The DNNs trained on unperturbed data generally have smaller *p*-values, except for the two most severe filters. This implies that the effect of filtering on microcalcifications can be attributed to information loss, and not solely to dataset shift. In other words, high frequency components in microcalcifications contain information that is important to the perception of DNNs.

Meanwhile, for soft tissue lesions (Fig. 4b), training on low-pass-filtered data significantly reduces the effect on predictive confidence and class separability, even for severe filters. This suggests that the effect of low-pass filtering on soft tissue lesions can primarily be attributed to dataset shift rather than information loss. In fact, DNNs trained with low-pass-filtered data maintain a similar level of class separability compared to networks trained on the unperturbed data. This confirms what we observed for radiologists, which is that high frequency components in soft tissue lesions are largely dispensable, and that more robust features exist.

### Annotation reader study

 Our analysis thus far has purely been in the frequency domain. Here, we extend our comparison to the spatial domain by examining the degree to which radiologists and DNNs agree on the most suspicious regions of an image. We conducted a reader study in which seven radiologists annotated up to three regions of interest (ROIs) containing the most suspicious features of each image. 120 exams were used in this study, which is a subset of the 720 exams in the perturbation reader study. See the Methods section for details regarding this reader study. We then applied low-pass filtering to the interior and exterior of the ROIs, as well as to the entire image. Examples of the annotation and the low-pass filtering schemes are shown in Fig. 2g–j. We made predictions using DNNs trained with the unperturbed data in order to understand the relationship between the high frequency components utilized by DNNs, and the regions of mammograms that are most suspicious to the radiologists.

### Comparing humans and machines with respect to the regions of an image deemed most suspicious

**Figure 5:**
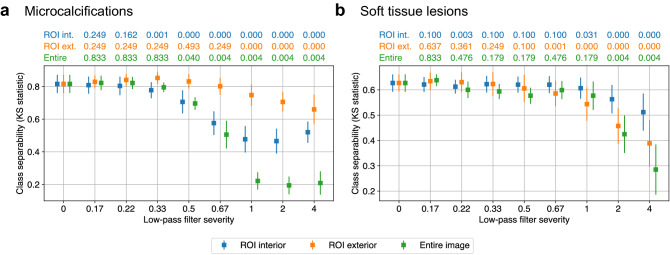
Comparing humans and machines with respect to the regions of an image deemed most suspicious. The performance of DNNs trained on unfiltered images was evaluated on images with selective perturbations in regions of interest (ROIs) identified as suspicious by human radiologists. **(a)** For microcalcifications, filtering the ROI interior decreases predictive confidence, but not as much as filtering the entire image. Filtering the ROI exterior decreases predictive confidence as well, meaning that DNNs utilize high frequency components in both the interior and the exterior of the ROIs, whereas humans focus more selectively on those ROIs. **(b)** For soft tissue lesions, filtering the ROI interior has very little effect on class separability. Meanwhile, filtering the ROI exterior has a similar effect to filtering the entire image. This implies that the high frequency components used by DNNs in these lesion subgroups are not localized in the areas that radiologists consider suspicious. Figure created with drawio v13.9.0 https://github.com/jgraph/drawio.

We began by comparing the effect of the three low-pass filtering schemes on the DNNs’ predictions for microcalcifications (Fig. 5a). We observed that filtering the ROI interior has a similar effect to filtering the entire image for mild filter severities. This suggests that for these frequencies, DNNs primarily rely on the same regions that radiologists consider suspicious. Meanwhile, class separability for the two ROI-based filtering schemes diverge significantly for high severities: filtering the ROI interior ceases to further decrease class separability at some threshold filter severity, whereas exterior filtering continues to degrade class separability beyond this threshold. The implication is that a range of high frequency components utilized by DNNs exist in the exterior of the ROIs deemed important by radiologists.

For soft tissue lesions (Fig. 5b), filtering the ROI interior decreases class separability, but to a lesser degree compared to filtering the entire image. This means that DNNs do utilize high frequency components in regions that radiologists find suspicious, but only to a limited degree. Meanwhile, filtering the ROI exterior has a similar effect on class separability as filtering the entire image. These observations suggest that the high frequency components that DNNs use for soft tissue lesions may be scattered across the image, rather than being localized in the areas that radiologists consider suspicious. While it is already established that DNNs utilize global information in screening mammograms^37^, this effect appears to be more pronounced for soft tissue lesions compared to microcalcifications.

## Discussion

**Figure 6:**
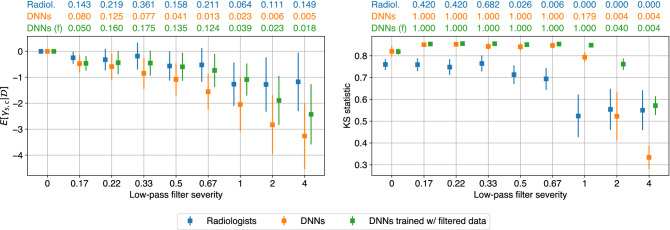
Simpson’s paradox leads to incorrect conclusions. If we merged microcalcifications and soft tissue lesions into a single subgroup, we would incorrectly conclude that radiologists and DNNs exhibit similar perturbation robustness both for predictive confidence (left) and for class separability (right). This highlights the importance of performing subgroup analysis when comparing human and machine perception. Figure created with drawio v13.9.0 https://github.com/jgraph/drawio.

Our framework draws inspiration from perturbation robustness studies in the adjacent domain of natural images, where there is also an ongoing crisis regarding the trustworthiness of machine perception. One key innovation in our work is to incorporate subgroup analysis to draw precise conclusions regarding perception. Not accounting for subgroups can be very dangerous, as it can lead to drawing erroneous conclusions due to Simpson’s paradox. In our case study, our conclusions would change significantly if we treated microcalcifications and soft tissue lesions as a single subgroup. As shown in Fig. 6, we would incorrectly conclude that radiologists and DNNs have comparable perturbation robustness in terms of both predictive confidence and class separability, thus artificially inflating the similarity between human and machine perception.

The identification of subgroups with clinically meaningful differences is a crucially important component of our framework, as it strongly influences the conclusions. It requires domain knowledge, and is not as simple as enumerating all possible subgroups. The reason is that, due to the rarity of some subgroups, there is a balance to strike between the number of specified subgroups and the amount of available data. In our case study, we combined architectural distortions, asymmetries, and masses into the soft tissue lesion subgroup because there are only 30 cases of malignant soft tissue lesions in our reader study dataset. By doing this, we addressed data scarcity while accounting for the fact that soft tissue lesions as a whole are much less localized than microcalcifications, and thus require a significantly different diagnostic approach.

The choice of input perturbation is another important consideration. For the purpose of understanding perception, it is more important for the removed information to be clearly-characterizable than for the perturbation to be clinically realistic. For example, analyzing robustness in cross-institutional settings is clinically realistic, but it does not allow us to draw precise conclusions regarding perception, since it is unclear what information may have changed between institutions. Having said that, if the perturbation removes clinically relevant information and is additionally clinically realistic, this is beneficial because it allows us to reason about robustness in a plausible scenario. Our choice of Gaussian low-pass filtering is clinically relevant, as another type of low-pass filtering called motion blurring does occur in practice. Mammograms can be blurred by motion caused by patients or imaging devices^38^, and^39^ demonstrated that it can degrade the ability of radiologists to detect malignant lesions. While there exist differences between Gaussian low-pass filtering and motion blurring, we expect that robustness to the former will translate to the latter. This is because DNNs have been shown to exhibit similar robustness characteristics to various types of blurring^27^. We noticed that when comparing the class separability between radiologists and DNNs trained with low-pass-filtered data (Fig. 4), DNNs are more robust to low-pass filtering for microcalcifications, while both are largely invariant for soft tissue lesions. This may be a significant advantage of DNNs in clinical practice.

It is nontrivial to ensure a fair comparison between humans and machines, and there is a growing body of work contemplating this issue. In^40^, the authors draw inspiration from cognitive science, and argue that we should compare humans and machines with respect to competence, rather than performance. One suggestion they make is to make humans similar to machines, or vice versa. As an example, consider the fact that the human visual system is foveated, meaning that incoming light is sampled with spatially-varying resolution. In contrast, machines typically perceive images at a uniform resolution. In^41^, the authors encode this inductive bias into a DNN architecture to demonstrate several advantages in generalization and robustness. The authors of^42^ also advocate for aligning experimental conditions between humans and machines in order to avoid drawing conclusions which are incorrect due to flawed experimental design. With these related works in mind, we made several considerations to ensure a fair comparison between humans and machines.

First, given the infeasibility of perfectly aligning experimental conditions, we did not directly compare radiologists and DNNs with respect to any evaluation metrics. In other words, we avoided drawing conclusions such as “radiologists are more robust than DNNs to low-pass filtering because their predictive confidence is higher.” Instead, we compared radiologists and DNNs to themselves in the unperturbed setting, and drew conclusions by contrasting how they changed when exposed to low-pass filtering.

Second, we made sure that none of the reported differences between radiologists and DNNs was due to the low-pass filtering being imperceptible to humans. The main difference we observe between radiologists and DNNs is that for soft tissue lesions, DNNs are sensitive to a range of high frequencies that humans are invariant to. This observed difference occurs at a range of high frequencies which, according to previous work, is perceptible to humans. We measure the severity of low-pass filtering as a wavelength in units of millimeters on the physical breast. Previous work^39^ showed that simulated image blurring is visible to radiologists from 0.4 mm of movement. We experimented with eight severities ranging from 0.17 to 4 mm. Of these, {0.17 mm, 0.2 mm, 0.33 mm} are below the visible threshold of 0.4 mm reported in^39^. Our conclusions remain in tact even if we were to remove these mild severities from our analysis.

Third, it is unclear to what degree low-pass filtering affects radiologists by removing salient information, versus making the images look different from what they are used to seeing. We minimized the latter effect by choosing a Gaussian filter which does not leave visible artifacts. Since it is impractical to retrain radiologists using low-pass filtered images, it is arguable which is a fairer comparison: DNNs trained on unperturbed images, or DNNs trained on low-pass filtered images. We therefore included results for both, and verified that our main conclusions do not depend on this choice.

Finally, we accounted for the fact that radiologists routinely zoom into suspicious regions of an image, and also simultaneously look at multiple images within an exam. Similar to^41^, we experimented with DNN architectures designed to mimic these behaviors. The GMIC architecture^13,14^ exhibits the zooming behavior, but only processes one image at a time. On the other hand, the DMV architecture^12^ does not zoom in, but processes multiple images simultaneously. All of our conclusions hold for both architectures, which suggests that our conclusions are not sensitive to either of these radiologists’ behaviors.

In summary, we proposed a framework for comparing human and machine perception in medical diagnosis, which we expect to be applicable to a variety of clinical tasks and imaging technologies. The framework uses perturbation robustness to compare human and machine perception. To avert Simpson’s paradox, we draw separate conclusions for subgroups that differ significantly in their diagnostic approach. We demonstrated the efficacy of this framework with a case study in breast cancer screening, and revealed significant differences between radiologists and DNNs. For microcalcifications, radiologists and DNNs are both sensitive to low-pass filtering, so we were unable to conclude whether they use different features. In contrast, radiologists are invariant and DNNs are sensitive to low-pass filtering on soft tissue lesions, which suggests that they use different features for this subgroup. From our annotation reader study, we found that these DNN-specific features in soft tissue lesions predominantly exist outside of the regions of an image found most suspicious by radiologists. We also showed that we would have missed this stark divergence between radiologists and DNNs in soft tissue lesions if we failed to perform subgroup analysis. This is evidence that future studies comparing human and machine perception in other medical domains should separately analyze subgroups with clinically meaningful differences. By utilizing appropriate subgroup analysis driven by clinical domain knowledge, we can draw precise conclusions regarding machine perception, and potentially accelerate the widespread adoption of DNNs in clinical practice.

## Methods

All methods were carried out in accordance with relevant guidelines and regulations, and consistent with the Declaration of Helsinki. The NYU Breast Cancer Screening Dataset^33^ was obtained under the NYU Langone Health IRB protocol ID#i18-00712_CR3. Informed consent was waived by the IRB. This dataset was extracted from the NYU Langone Health private database, and is not publicly available.

### DNN training methodology

 We conducted our DNN experiments using two architectures: the Deep Multi-View Classifer^12^, and the Globally-Aware Multiple Instance Classifier^13,14^. With both architectures, we trained an ensemble of five models. A subset of each model’s weights was initialized using weights pretrained on the BI-RADS label optimization task^43^, while the remaining weights were randomly initialized. For each architecture, we adopted the same training methodology used by the corresponding authors.

### Probability calibration

 We applied Dirichlet calibration^44^ to the predictions of DNNs used in our probabilistic modeling. This amounts to using logistic regression to fit the log predictions to the targets. We trained the logistic regression model using the validation set, and applied it to the log predictions on the test set to obtain the predictions used in our analysis. We used L2 regularization when fitting the logistic regression model, and tuned the regularization hyperparameter via an internal 10-fold cross-validation where we further split the validation set into “training” and “validation” sets. In the cross-validation, we minimized the classwise expected calibration error^44^.

### Gaussian low-pass filtering

 Low-pass filtering is a method for removing information from images that allows us to interpolate between the original image and, in the most extreme case, an image where every pixel has the value of the mean pixel value of the original image. We experimented with nine filter severities selected to span a large range of the frequency spectrum. We implemented the Gaussian low-pass filter by first applying the shifted two-dimensional discrete Fourier transform to transform images to the frequency domain. The images were multiplied element-wise by a mask with values in [0, 1]. The values of this mask are given by the Gaussian function

2 $$\begin{aligned} M(u, v) = \exp \left( \frac{-D^2(u, v)}{2D^2_0}\right) , \end{aligned}$$

where *u* and *v* are horizontal and vertical coordinates, *D*(*u*, *v*) is the Euclidian distance from the origin, and $D_0$ is the cutoff frequency. $D_0$ represents the severity of the filter, where frequencies are reduced to 0.607 of their original values when $D(u, v) = D_0$. Since the mammograms in our dataset vary in terms of spatial resolution as well as the physical length represented by each pixel, we expressed the filter severity $D_0$ in terms of a normalized unit of cycles per millimeter on the breast. Let $\alpha = \min (H, W)$ where *H* and *W* are the height and width of the image, and let $\beta$ denote the physical length in millimeters represented by each pixel. Then we can convert cycles per millimeter $D_0$ to cycles per frame length of the image $D_0^{\text {img}}$ using

3 $$\begin{aligned} D_0^{\text {img}} = D_0 \cdot \alpha \cdot \beta . \end{aligned}$$

### Perturbation reader study 

In order to compare humans and machines with respect to their perturbation robustness, we conducted a reader study in which ten radiologists read 720 exams selected from the test set. While all radiologists read the same set of exams, each exam was low-pass filtered with a different severity for each radiologist. Except for the low-pass filtering, the setup of this reader study is identical to that of^12^, and it was up to the radiologists to decide what equipment and techniques to use. Each exam consists of at least four images, with one or more images for each of the four views of mammography: L-CC, R-CC, L-MLO, and R-MLO. All images in the exam were concatenated into a single image such that the right breast faces left and is presented on the left, and the left breast faces right and is displayed on the right. Additionally, the craniocaudal (CC) views are on the top row, while the mediolateral oblique (MLO) views are on the bottom row. An example of this is shown in Supplementary Information Fig. 1. Among the 1440 breasts, 62 are malignant, 356 are benign, and the remaining 1022 are nonbiopsied. Among the malignant breasts, there are 26 microcalcifications, 21 masses, 12 asymmetries, and 4 architectural distortions, while in the benign breasts, the corresponding counts are: 102, 87, 36, and 6. For each exam, radiologists make a binary prediction for each breast, indicating their diagnosis of malignancy.

### Probabilistic modeling

 We modeled the radiologists’ and DNNs’ binary malignancy predictions with the Bernoulli distribution

4 $$\begin{aligned} {\hat{y}}_{r, s}^{(n)} \sim {{\,\mathrm{Bernoulli}\,}}(p_{r, s}^{(n)}), \end{aligned}$$

where $n \in \{1, 2, \dotsc , 1440\}$ indexes the breast, $r \in \{1, 2, \dotsc , 10\}$ the reader, and $s \in \{1, 2, \dotsc , 9\}$ the low-pass filter severity. Each distribution’s parameter $p_{r, s}^{(n)}$ is a function of four latent variables

$$\begin{aligned} p_{r, s}^{(n)} = \sigma (b_g + \mu ^{(n)} + \gamma _{s, g} + \nu _{r, g}), \end{aligned}$$

where $c \in \{1, \dotsc , 5\}$ indexes the subgroup of the lesion. We included the following subgroups: unambiguous microcalcifications, unambiguous soft tissue lesions, ambiguous microcalcifications and soft tissue lesions, mammographically occult, and nonbiopsied. We considered these five subgroups in order to make use of all of our data, but only used the first two in our analysis. We assigned the generic weakly informative prior ${\mathcal {N}}(0, 1)$ to each latent variable. We chose the Bernoulli distribution because it has a single parameter, and thus makes the latent variables interpretable. Additionally, radiologists are accustomed to making discrete predictions in clinical practice. The posterior distribution of the latent variables is given by

$$\begin{aligned} p({\varvec{b}}, \varvec{\mu }, \varvec{\gamma }, \varvec{\nu } \mid \varvec{{\hat{y}}}) = \frac{p(\varvec{{\hat{y}}} \mid {\varvec{b}}, \varvec{\mu }, \varvec{\gamma }, \varvec{\nu })}{p\left( \varvec{{\hat{y}}}\right) }. \end{aligned}$$

The exact computation of the posterior is intractable, since the marginal likelihood $p(\varvec{{\hat{y}}})$ involves a four-dimensional integral. We therefore applied automatic differentiation variational inference (ADVI)^45^ in order to approximate the posterior. ADVI, and variational inference in general, optimizes over a class of tractable distributions in order to find the closest match to the posterior. For our choice of tractable distributions, we used the mean-field approximation, meaning that we optimized over multivariate Gaussians with diagonal covariance matrices.

In practice, while radiologists made binary predictions, DNNs made continuous predictions in [0, 1] that we then calibrated. Despite the DNN predictions not being binary, we used equivalent procedures to specify the probabilistic model for radiologists and DNNs. To see how, let ${\hat{y}}^{(n)} \in \{0, 1\}$ denote a DNN’s unobserved binary prediction for case *n*, and let ${\hat{z}}^{(n)} \in [0, 1]$ denote its observed real-valued prediction for the same case. In order to obtain ${\hat{y}}^{(n)} \in \{0, 1\}$, we could treat it as a random variable ${\hat{y}}^{(n)} \sim {{\,\mathrm{Bernoulli}\,}}({\hat{z}}^{(n)})$ and obtain values for it through sampling. We instead used ${\hat{z}}^{(n)}$ directly, specifying the log joint density as

$$\begin{aligned} \begin{aligned} \log p(\varvec{{\hat{y}}} \mid \theta )&= \sum _{n=1}^{N} \log p({\hat{y}}^{(n)} \mid \theta ^{(n)})\\&\approx \sum _{n=1}^{N} {\mathbb {E}}_{{\hat{y}}^{(n)}}[\log p({\hat{y}}^{(n)} \mid \theta ^{(n)})]\\&= \sum _{n=1}^{N} {\mathbb {E}}_{{\hat{y}}^{(n)}}\left[ {\hat{y}}^{(n)} \log (\theta ^{(n)}) + (1 - {\hat{y}}^{(n)}) \log (1 - \theta ^{(n)})\right] \\&= \sum _{n=1}^{N} \Pr ({\hat{y}}^{(n)} = 1) \log (\theta ^{(n)}) + \Pr ({\hat{y}}^{(n)} = 0) \log (1 - \theta ^{(n)})\\&= \sum _{n=1}^{N} {\hat{z}}^{(n)} \log (\theta ^{(n)}) + (1 - {\hat{z}}^{(n)}) \log (1 - \theta ^{(n)}). \end{aligned} \end{aligned}$$

### Annotation reader study

In order to compare humans and machines with respect to the regions of an image deemed most suspicious, we conducted a reader study in which seven radiologists read the same set of 120 unperturbed exams. The exams in this study were a subset of the 720 exams from the perturbation reader study, and also included all malignant exams from the test set. This study had two stages. In the first stage, the radiologists were presented with all views of the mammogram, and they made a malignancy diagnosis for each breast. This stage was identical to the reader study in^12^. In the second stage, for breasts that were diagnosed as malignant, the radiologists annotated up to three ROIs around the regions they found most suspicious. Overlapping ROIs were permitted. The radiologists annotated each view individually, and the limit of three ROIs applied separately to each view. For exams that contained multiple images per view, the radiologists annotated the image where the malignancy was most visible. The radiologists annotated the images using Paintbrush on MacOS, or Microsoft Paint on Windows. In order to constrain the maximum area that is annotated for each image, we included a $250{\times }250$ pixel blue ROI template in the bottom corner of each image to serve as a reference. The radiologists then drew up to three red ROIs such that each box approximately matched the dimensions of the reference blue ROI template. In our subsequent analysis, we computed results individually using each radiologist’s ROIs, and then reported the mean and standard deviation across radiologists

## Supplementary Information

Supplementary Information.

## Data Availability

The radiologist and DNN predictions used in our analysis are available at https://github.com/nyukat/perception_comparison under the GNU AGPLv3 license.

## Code availability

The code used in this research is available at https://github.com/nyukat/perception_comparison under the GNU AGPLv3 license. This, combined with the predictions which we also open-sourced, makes our results fully reproducible. We used several open-source libraries to conduct our experiments. The DNN experiments were performed using PyTorch^46^, and the probabilistic modeling was done with PyStan (https://github.com/stan-dev/pystan), the Python interface to Stan^47^. The code for the DNNs used in our experiments is also open-source, where GMIC is available at https://github.com/nyukat/GMIC, and DMV at https://github.com/nyukat/breast_cancer_classifier.
