# Supplementary material for: Differences between human and machine perception in medical diagnosis
Source: Sci Rep. 2022 Apr 27;12:6877. doi: 10.1038/s41598-022-10526-z (PMC9046399; doi:10.1038/s41598-022-10526-z)
Supplement: Supplementary file 1 — Supplementary Information. [file 41598_2022_10526_MOESM1_ESM.pdf]

Differences between human and machine perception in medical diagnosis (supplementary information)

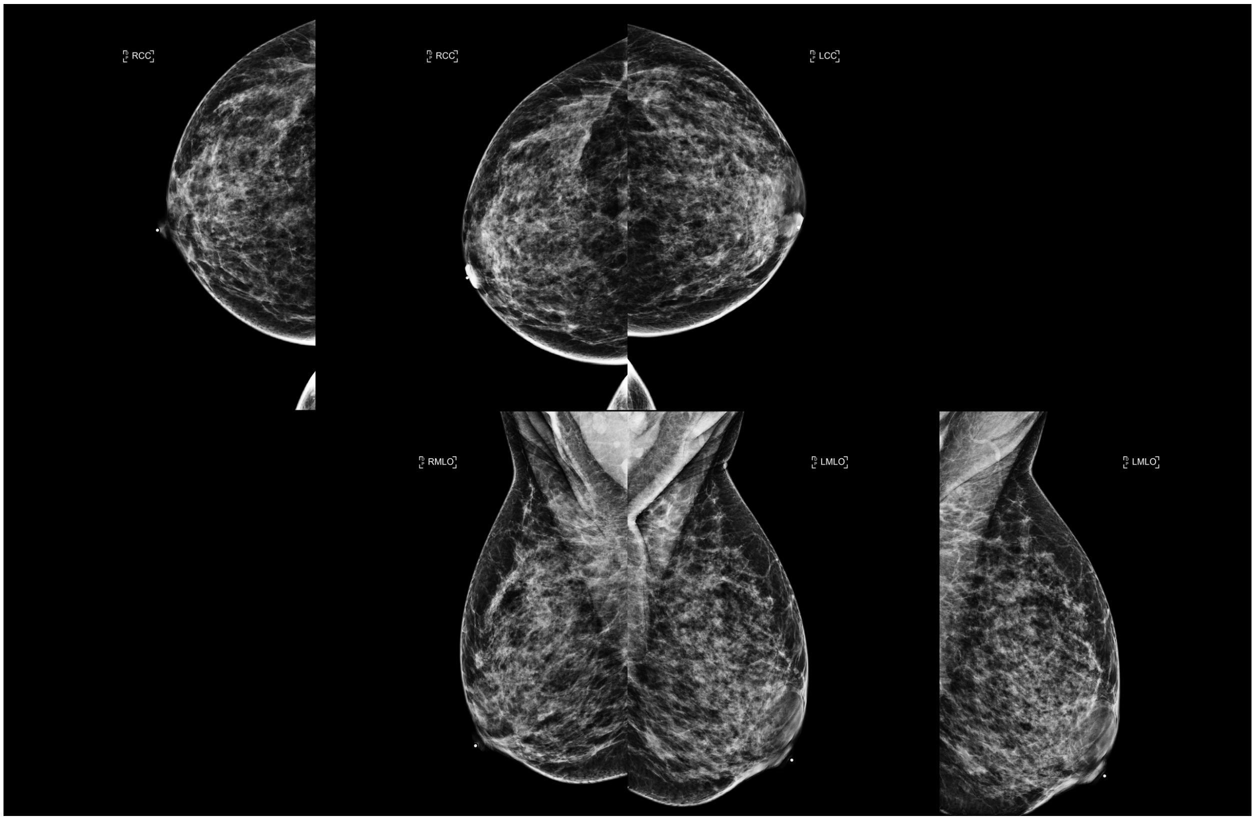

Supplementary Figure 1: **An example of a screening mammogram.** An exam is composed of at least one image from each of four views. The views, in clockwise order, are: left craniocaudal (L-CC), left mediolateral oblique (L-MLO), right mediolateral oblique (R-MLO), and right craniocaudal (R-CC). This example shows that there can be multiple images per view. We present the images to radiologists in a format called ventral hanging, where the right breast faces left and is presented on the left, and the left breast faces right and is displayed on the right. Additionally, the craniocaudal (CC) views are on the top row, while the mediolateral oblique (MLO) views are on the bottom row. In contrast to radiologists, DNNs made predictions on each image individually after they were cropped to a consistent size. Figure created with drawio v13.9.0 `https://github.com/jgraph/drawio`.

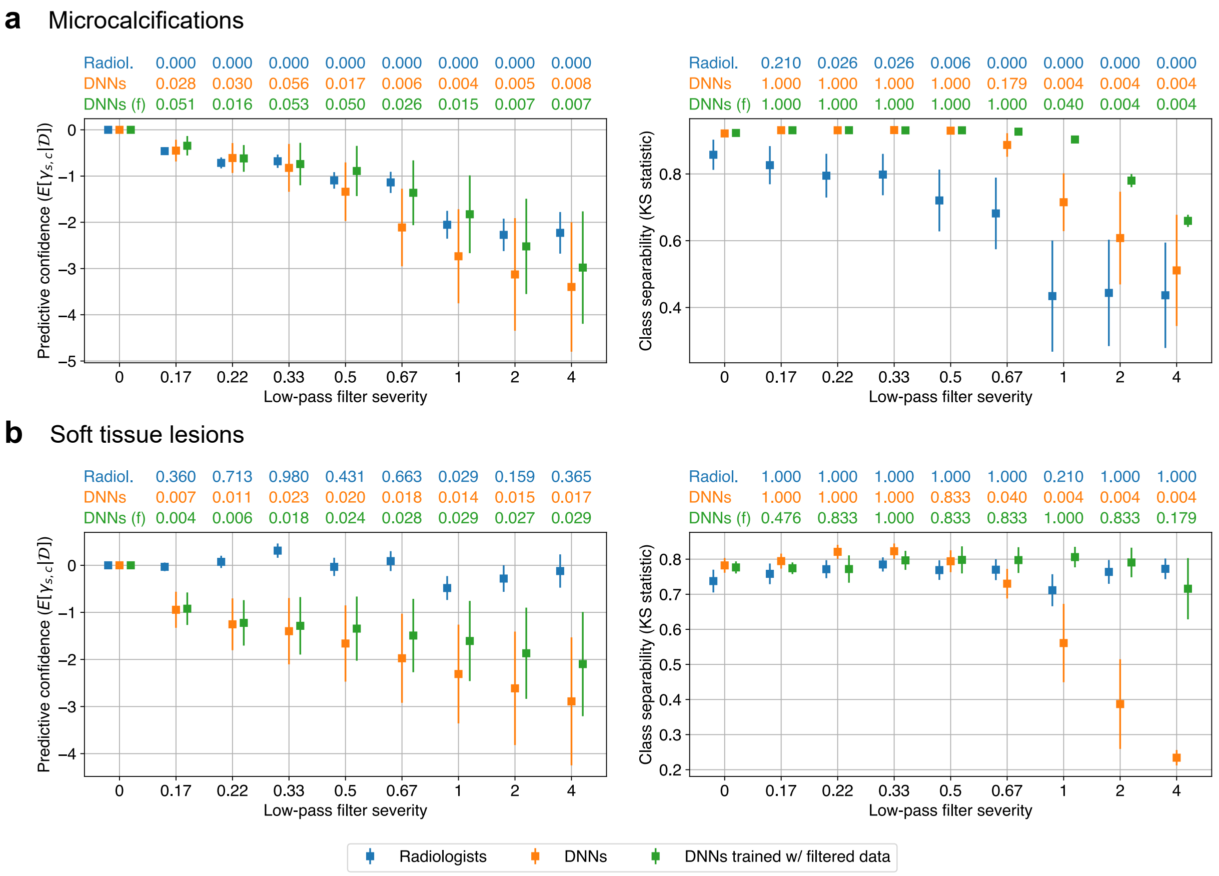

Supplementary Figure 2: **Comparing humans and machines with respect to their perturbation robustness.** These results are for the DMV architecture, and our conclusions are the same as with the GMIC architecture. Figure created with drawio v13.9.0 `https://github.com/jgraph/drawio`.

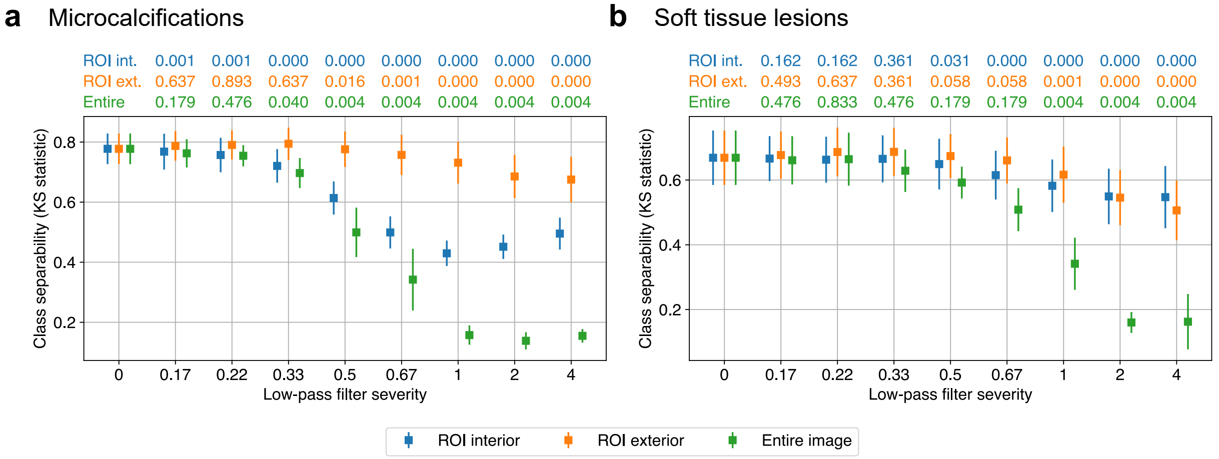

Supplementary Figure 3: **Comparing humans and machines with respect to the regions of an image deemed most suspicious.** These results are for the DMV architecture, and our conclusions are the same as with the GMIC architecture. Figure created with drawio v13.9.0 `https://github.com/jgraph/drawio`.
